# Epidemiological and characteristic differences of hypervirulent and classical *Klebsiella pneumoniae*: a clinical and genomic study in Southern China during the COVID-19 pandemic

**DOI:** 10.3389/fcimb.2025.1701929

**Published:** 2026-01-16

**Authors:** Yushan Jiang, Nianqing Kong, Zhuolin Li, Weiling Wu, Yifei Xie, Zhujun Zeng, Tingting Peng, Chenguang Shen, Shi Ouyang

**Affiliations:** 1 Department of Infectious Diseases, Key Laboratory of Biological Targeting Diagnosis, Therapy and Rehabilitation of Guangdong Higher Education Institutes, The Fifth Affiliated Hospital, Guangzhou Medical University, Guangzhou, China; 2 BSL-3 Laboratory (Guangdong), Guangdong Provincial Key Laboratory of Tropical Disease Research, School of Public Health, Southern Medical University, Guangzhou, China; 3 Department of Laboratory Medicine, Baiyun District People's Hospital of Guangzhou, Guangzhou, China; 4 Medical Laboratory Department, Guangdong Provincial Hospital of Chinese Medicine, Zhuhai, China; 5 Department of Laboratory Medicine, Zhujiang Hospital, Key Laboratory of Infectious Diseases Research in South China, Southern Medical University, Ministry of Education, Guangzhou, China

**Keywords:** community-acquired infection, drug resistance, hypervirulence, *Klebsiella pneumoniae*, multilocus sequence typing

## Abstract

**Introduction:**

Hypervirulent *Klebsiella pneumoniae* (hvKp) has emerged as a significant public health threat owing to its ability to cause invasive infections. This study aimed to investigate the clinical characteristics and epidemiological associations of hypervirulent Klebsiella pneumoniae (hvKp) and classical *K. pneumoniae* (cKp) among patients treated at a tertiary hospital in Zhuhai City, Guangdong Province, China, during the period from January to December 2022, in the context of the ongoing COVID-19 pandemic.

**Method:**

A total of 97 non-duplicated *K. pneumoniae* isolates and corresponding clinical data were collected. Antimicrobial susceptibility testing, hypermucoviscosity phenotyping, sequence typing, capsular serotyping, and whole-genome sequencing were performed. Hypervirulent strains were identified by the presence of the *rmpA*, *rmpA2*, *iucA*, *iroB*, *peg-344*, and *peg-589* genes.

**Results:**

Among the 97 isolates, 40 (41.2%) were classified as hvKp. Compared with cKp, hvKp was significantly more likely to cause bacteraemia (*P* < 0.05) and less likely to cause urinary tract infections (*P* < 0.05). The K20 capsular serotype was significantly associated with hvKp isolates (*P* < 0.05). The multidrug resistance rate among hvKp strains (22.5%) was markedly lower than that among cKp strains (56.63%), and extended-spectrum β-lactamase production was more common in cKp strains. Multilocus sequence typing identified 29 sequence types, including 24 novel types. Whole-genome sequencing of a multidrug-resistant hvKp isolate (Kp00198874) revealed an ST11-K64 strain resistant to all tested antibiotics.

**Discussion:**

The prevalence of hvKp increased during the COVID-19 pandemic in Guangdong, China. The isolates identified in this study represent sporadic infections, and the emergence of ST11-K64 hypervirulent carbapenem-resistant K. pneumoniae (hv-CRKp) highlights the urgent need for continued surveillance and vigilance regarding hvKp-associated bacteraemia.

## 1 Introduction

*Klebsiella pneumoniae* is a clinically significant gram-negative bacterium recognized as the second most common cause of community-acquired infections in Asia (Magill et al., 2018; Hwang et al., 2020). As a major healthcare-associated pathogen, *K. pneumoniae* is frequently responsible for urinary tract, bloodstream, and lung infections, with purulent abscesses at various sites also being common (Zhu et al., 2025). The proportion of *Klebsiella* bacteremia cases attributed to community-acquired infections ranged from 25% to 50% as early as the 1980s (García de la Torre et al., 1985; Wang et al., 1990). Notably, a distinct clinical syndrome characterized by community-acquired *K. pneumoniae* bacteremia, accompanied by primary liver abscess, metastatic meningitis, and endophthalmitis, was first identified in Taiwan (Ko et al., 2002; Yu et al., 2006). However, there remains a lack of comprehensive studies comparing the clinical and molecular characteristics of hypervirulent *K. pneumoniae* (hvKp) and classical *K. pneumoniae* (cKp) strains in Southern China, particularly in the context of the COVID-19 pandemic.

*K. pneumoniae* is also one of the “ESKAPE” pathogens, a group of organisms known for their ability to evade the biocidal action of antibiotics and represent emerging paradigms in pathogenesis, transmission, and drug resistance (Tarasenko et al., 2025). This bacterium is notorious for accumulating and rapidly disseminating multidrug resistance (MDR) determinants (Goodarzi et al., 2025). Zhang et al. reported an MDR rate of 8.05% among *K. pneumoniae* isolates collected from a hospital in Beijing, China, between 2007 and 2009 (Zhang et al., 2012). However, owing to the overuse of antibiotics and the spread of resistance plasmids, the MDR rate has steadily increased, with reports in China ranging from 45% to 61% (Li et al., 2012; Liu et al., 2020; Le et al., 2021). These findings highlight the need for continuous monitoring of *K. pneumoniae* infections and resistance trends.

*K. pneumoniae* has evolved into two distinct pathotypes: hvKp and cKp. Unlike cKp, hvKp can cause invasive infections even in healthy individuals, leading to spontaneous abscess formation, particularly liver abscesses, and the development of metastatic infections, with or without secondary bacteremia (Ma et al., 2025) Among more than 79 described capsular serotypes, K1, K2, K5, K20, K54, and K57 are strongly associated with the invasiveness and pathogenicity of hvKp (Shon et al., 2013; Li et al., 2022). Despite extensive research, a consensus definition for hvKp remains elusive (Zhu et al., 2025). Recent studies have proposed that virulence genes such as *rmpA*, *rmpA2*, *iucA*, *iroB*, and *peg-344* may serve as more reliable markers for identifying hypervirulence than traditional methods such as the string test or capsular serotyping (Russo et al., 2018). Although hvKp strains have generally been susceptible to conventional antibiotics (Cai et al., 2023), the emergence of multidrug-resistant hypervirulent strains, resulting from MDR cKp strains acquiring pLVPK-like virulence plasmids, has led to a fatal nosocomial outbreak (Gu et al., 2018). The convergence of hypervirulence and multidrug resistance in *K. pneumoniae* represents a serious public health threat (Yang et al., 2023), underscoring the urgent need for strategies to prevent and control the spread of MDR-hvKp. In addition, although the clinical and molecular characteristics of hvKp and cKp have been compared across various provinces in China, reports from Guangdong Province remain limited.

Therefore, this study analyzed 97 K*. pneumoniae* isolates collected from a tertiary hospital in Zhuhai City, Guangdong Province, China, to fully understand the clinical and molecular characteristics of hvKp and cKp during the COVID-19 pandemic. This study aimed to compare the clinical characteristics, prevalence, and antibiotic resistance patterns of hvKp and cKp strains. In addition, whole-genome sequencing of a hypervirulent carbapenem-resistant Kp (hv-CRKp) isolate (ST11-K64) was performed. These findings provide critical molecular and clinical insights into hvKp and offer important information to guide the clinical management of hvKp infections.

## 2 Methods

### 2.1 Collection and identification of *K. pneumoniae* clinical isolates

A total of 97 non-duplicated *K. pneumoniae* isolates associated with community-acquired infections were collected during the COVID-19 era (from January 2022 to December 2022) from 80 patients at a tertiary hospital in Zhuhai City, Guangdong Province, China. Isolates were identified using a VITEK-32 microbiological analyzer (bioMérieux, Marcy-l’Étoile, France) and stored at −80 °C in Lysogeny broth (LB) supplemented with 20% glycerol (v/v) until further analysis.

### 2.2 Patient information

Clinical information, including sex, age, and type of infection (lung, urinary tract, bloodstream, skin, abdominal, biliary tract, and perianal infections and liver abscess), was collected from the hospital’s clinical laboratory database to analyze the clinical characteristics of patients with *K. pneumoniae* infections.

### 2.3 String test assays

The string test was performed to detect the hypermucoviscous phenotype as previously described (Liu and Guo, 2018). *K. pneumoniae* isolates were subcultured on LB broth agar medium and incubated overnight at 37°C. An inoculation loop was placed on the surface of single colonies, and it was observed whether a string formed when the loop was drawn slowly away from the colonies. Formation of a mucoviscous string ≥5 mm was defined as a positive test.

### 2.4 Antimicrobial susceptibility test

Antimicrobial susceptibility testing was performed using the VITEK-2 automated platform (bioMérieux). Antimicrobial agents tested included piperacillin–tazobactam (TZP), cefazolin (CZO), cefotetan (CTT), ceftazidime (CAZ), ceftriaxone (CRO), cefepime (FEP), ertapenem (ETP), imipenem (IPM), amikacin (AMK), gentamicin (GEN), tobramycin (TOB), ciprofloxacin (CIP), levofloxacin (LEV), nitrofurantoin (F), trimethoprim–sulfamethoxazole (SXT), ampicillin–sulbactam (SAM), and aztreonam (ATM).

Multidrug resistance was defined as resistance to three or more antimicrobial classes. Extended-spectrum β-lactamase (ESBL) production was detected using the VITEK-2 system. Results were interpreted according to the Clinical and Laboratory Standards Institute guidelines 2023 (CLSI, 2023).

### 2.5 Detection of virulence factors

Hypervirulent *K. pneumoniae* was defined by the presence of at least two of the following plasmid-borne virulence genes, as described previously: *rmpA*, *rmpA2*, *iucA*, *iroB*, *peg-344*, and *peg-589* (Russo et al., 2018). Plasmid DNA was extracted using the TIANprep Rapid Mini Plasmid Kit [Tiangen Biotech (Beijing) Co., Ltd., Beijing, China]. Polymerase chain reactions (PCRs) (50 μL) contained 5 ng of plasmid DNA, 25 μL of Green Taq Mix (Vazyme, Nanjing, China), and 10 μM of each primer (forward and reverse). Specific primer sequences, expected product sizes, and PCR conditions are listed in **Supplementary Table S1**.

### 2.6 Detection of capsular serotype

Capsular serotypes K1, K2, K5, K20, K54, and K57 were detected via PCR amplification of serotype-specific genes as previously described (Yu et al., 2008). Each PCR (50 μL) included 1 μL of crude DNA extract (obtained by centrifuging bacterial suspensions and using the supernatant), 25 μL of Green Taq Mix (Vazyme), and 10 μM of each primer. PCR products were separated via electrophoresis on 1.5% (w/v) agarose gels, and fragment sizes were compared with the DL2000 DNA marker (Takara Bio Inc., Shiga, Japan). Specific primer sequences, expected product sizes, and PCR conditions are listed in **Supplementary Table S1**.

### 2.7 Whole-genome sequencing (WGS) and bioinformatics analysis

Comprehensive antimicrobial susceptibility profiling and molecular characterization revealed that the MDR strain Kp00198874 was resistant to all tested antimicrobial agents and was subsequently selected for whole-genome sequencing (WGS). Genomic DNA was extracted and sequenced using the NovaSeq 6000 Sequencing platform (Illumina, San Diego, CA, USA) with paired-end library construction (average insert size, 150 bp). Raw data were processed using fastp, and assembly was performed using Unicycler with default settings. Plasmid sequences were identified via BLAST. Capsular typing was determined using K-PAM (https://www.iith.ac.in/K-PAM/pred_sertp.php). Virulence determinants were identified using the Virulence Factor Database (VFDB) (http://www.mgc.ac.cn/VFs/main.htm), and antimicrobial resistance genes were detected using the Comprehensive Antibiotic Resistance Database (CARD) (https://card.memaster.ea/). Plasmid comparisons were visualized using the BLAST Ring Image Generator.

### 2.8 Multilocus sequence typing

Overall, 19 cKp strains and 15 hvKp strains (including Kp00198874) were randomly selected for multilocus sequence typing (MLST). MLST was performed according to the protocol available on the Institut Pasteur’s Klebsiella MLST website (https://bigsdb.pasteur.fr/klebsiella/). Seven housekeeping genes (*gapA*, *mdh*, *phoE*, *tonB*, *infB*, *pgi*, and *rpoB*) were amplified (web-only **Supplementary Table S1**). Allelic profiles and sequence types were determined using data from the same website. To assess phylogenetic relationships among the selected strains, a maximum likelihood tree was constructed based on the concatenated sequences of the seven housekeeping genes using MEGA 11. The housekeeping gene sequences from the reference strain NTUH-K2044 (accession no. AP006725.1) were used as a reference for the phylogenetic analysis.

### 2.9 Statistical analysis

Statistical analyses were performed using the SPSS 24 software. Categorical variables were compared using the chi-square test or Fisher’s exact test, and continuous variables were compared using Student’s t-test. A *p*-value of <0.05 was considered statistically significant, and all probability values were two-tailed.

### 2.10 Nucleotide sequence accession numbers

The chromosome and plasmid sequences of Kp00198874 were deposited in the National Center for Biotechnology Information (NCBI) under BioProject accession number PRJNA1248222 and BioSample number SAMN51288166.

### 2.11 Ethics statement

The work described has been conducted in accordance with The Code of Ethics of the World Medical Association (Declaration of Helsinki) for experiments involving humans, and the studies were reviewed and approved by the ethics committee of The Fifth Affiliated Hospital of Guangzhou Medical University (GYWY-K2024-30).

## 3 Results

### 3.1 Characteristics of *K. pneumoniae* associated with community-acquired infections

The 97 *K. pneumoniae* isolates were obtained from 62 men and 35 women, with a median age of 70 years [interquartile range (IQR): 54–79 years]. Clinical characteristics are presented in **Table 1**. Most cases were lung infections (55/97, 56.7%), followed by urinary tract infections (30/97, 30.9%), bloodstream infections (8/97, 8.2%), and liver abscesses (2/97, 2.1%). Other infection types included skin, abdominal, and biliary tract infections (each 2.1%) and perianal infections (1/97, 1.0%).

**Table 1:** Clinical characteristics for *Klebsiella pneumoniae*, hvKp, and cKp.

Clinical characteristic	*K. pneumoniae* (n = 97)	hvKp (n = 40)	cKp (n = 57)	*p*-Value^a^
Age, years	70.00 (54.00-79.00)	70.00 (53.00-75.00)	68.00 (54.00-79.00)	0.821
Gender				
Male	62 (63.92%)	28 (70.00%)	34 (59.65%)	0.296
Female	35 (36.08%)	12 (30.00%)	23 (40.35%)	
Disease				
Lung infection	55 (56.70%)	25 (62.50%)	30 (52.63%)	0.334
Urinary tract infection	30 (30.93%)	10 (25.00%)	20 (35.09%)	0.006**
Bacteremia	8 (8.25%)	6 (15.00%)	2 (3.51%)	0.042*
Liver abscess	2 (2.06%)	0 (0.00%)	2 (3.51%)	0.231
Skin infection	2 (2.06%)	0 (0.00%)	2 (3.51%)	0.231
Abdominal infection	2 (2.06%)	0 (0.00%)	2 (3.51%)	0.231
Biliary tract infection	2 (2.06%)	0 (0.00%)	2 (3.51%)	0.231
Perianal infection	1 (2.03%)	1 (2.50%)	0 (0.00%)	0.230

hvKp, hypervirulent *K. pneumoniae*; cKp, classical *K. pneumoniae*.

a *p*-Value: hvKp vs. cKp. Significant differences were detected using the chi-square test or Fisher’s exact test; *p < 0.05; **p < 0.01; ***p < 0.001.

The distribution of virulence factors is summarized in **Table 2**. A hypermucoviscous phenotype was observed in 43.3% of isolates. Among the virulence genes, *iroB* was the most frequently detected (47.4%), followed by *iucA* (34.1%), *peg-589* (26.8%), *peg-344* (24.7%), and *rmpA* and *rmpA2* (each 4.1%). The K2 serotype was the most prevalent (16.5%, 16/97), followed by K20 and K57 (13.4% each). K54, K1, and K5 were infrequent, identified in 4, 3, and 1 isolates, respectively.

**Table 2:** Virulence genes and capsular serotypes for *Klebsiella pneumoniae*, hvKp, and cKp.

Virulence	*K. pneumoniae* (n = 97)	hvKp (n = 40)	cKp (n = 57)	*p*-Value^a^
String test	42 (43.30%)	31 (77.50%)	11 (19.30%)	<0.001***
*_p_rmpA*	4 (4.12%)	4 (10.00%)	0 (0.00%)	0.0147*
*_p_rmpA2*	4 (4.12%)	4 (10.00%)	0 (0.00%)	0.0147*
*iucA-1*	35 (36.08%)	30 (75.00%)	5 (8.77%)	<0.001***
*iroB-1*	46 (47.42%)	38 (95.00%)	8 (14.04%)	<0.001***
*peg-344*	24 (24.74%)	24 (60.00%)	0 (0.00%)	<0.001***
*peg-589-1*	26 (26.80%)	22 (55.00%)	4 (7.02%)	<0.001***
K1	3 (3.09%)	2 (5.00%)	1 (1.75%)	0.363
K2	16 (16.49%)	9 (22.50%)	7 (12.28%)	0.182
K5	1 (1.03%)	0 (0.00%)	1 (1.75%)	0.399
K20	13 (13.40%)	9 (22.50%)	4 (7.02%)	0.027*
K54	4 (4.12%)	1 (2.50%)	3 (5.26%)	0.500
K57	13 (13.40%)	4 (10.00%)	9 (15.79%)	0.410

hvKp, hypervirulent *K. pneumoniae*; cKp, classical *K. pneumoniae*.

a *p*-Value: hvKp vs. cKp. Significant differences were detected using the chi-square test or Fisher’s exact test; *p < 0.05; **p < 0.01; ***p < 0.001.

Antimicrobial susceptibility results are presented in **Table 3**. Of the 97 isolates, 39 (40.2%) were MDR, and 21 (21.6%) were ESBL producers. Among the 17 antibiotics tested, the highest resistance rates were observed in SAM (42.3%), CZO (40.2%), CIP (37.1%), CRO (35.1%), and both F and SXT (30.9%). Lower resistance was observed in LEV (23.7%), ATM (22.7%), CAZ (20.6%), GEN (13.4%), and both FEP and TZP (10.3%). Resistance was the lowest in CTT (9.3%), TOB (7.2%), IPM (5.2%), ETP (4.1%), and AMK (3.1%).

**Table 3:** Antimicrobial resistance profiles for *Klebsiella pneumoniae*, hvKp, and cKp.

Antimicrobial susceptibility ^b^	*K. pneumoniae* (n = 97)	hvKp (n = 40)	cKp (n = 57)	*p*-Value^a^
MDR	39 (40.21%)	9 (22.50%)	30 (52.63%)	0.003**
ESBL	21 (21.65%)	3 (7.50%)	18 (31.58%)	0.005**
TZP	10 (10.31%)	5 (12.50%)	5 (8.77%)	0.552
CZO	39 (40.21%)	8 (20.00%)	31 (54.39%)	<0.001***
CTT	9 (9.27%)	4 (10.00%)	5 (8.77%)	0.837
CAZ	20 (20.62%)	7 (17.50%)	13 (22.81%)	0.524
CRO	34 (35.05%)	8 (20.00%)	26 (45.61%)	0.009**
FEP	10 (10.31%)	5 (12.50%)	5 (8.77%)	0.552
ETP	4 (4.12%)	3 (7.50%)	1 (1.75%)	0.161
IPM	5 (5.15%)	3 (7.50%)	2 (35.09%)	0.381
AMK	3 (3.09%)	2 (5.00%)	1 (1.75%)	0.363
GEN	13 (13.40%)	4 (10.00%)	9 (15.79%)	0.409
TOB	7 (7.21%)	3 (7.50%)	4 (7.02%)	0.927
CIP	36 (37.11%)	10 (25.00%)	26 (45.61%)	0.038*
LEV	23 (23.71%)	4 (10.00%)	19 (33.33%)	0.007**
F	30 (30.93%)	11 (27.50%)	19 (33.33%)	0.541
SXT	30 (30.93%)	9 (22.50%)	21 (36.84%)	0.132
SAM	41 (42.27%)	10 (25.00%)	31 (54.39%)	0.003**
ATM	22 (22.68%)	7 (17.50%)	15 (26.32%)	0.307

a *p*-Value: hvKp vs. cKp. Significant differences were detected using the chi-square test or Fisher’s exact test; *p < 0.05; **p < 0.01; ***p < 0.001.

b Abbreviations: MDR, multidrug resistance; ESBL, extended-spectrum β-lactamase; TZP, piperacillin–tazobactam; CZO, cefazolin; CTT, cefotetan; CAZ, ceftazidime; CRO, ceftriaxone; FEP, cefepime; ETP, ertapenem; IPM, imipenem; AMK, amikacin; GEN, gentamicin; TOB, tobramycin; CIP, ciprofloxacin; LEV, levofloxacin; F, nitrofurantoin, SXT, trimethoprim–sulfamethoxazole; SAM, ampicillin–sulbactam; ATM, aztreonam; hvKp, hypervirulent *K. pneumoniae*; cKp, classical *K. pneumoniae*.

### 3.2 Virulence gene detection and classification of hvKp and cKp

Hypervirulent *K. pneumoniae* strains were identified by the presence of at least two of six key virulence genes (*rmpA*, *rmpA2*, *iucA*, *iroB*, *peg-344*, and *peg-589*) (Harada and Doi, 2018). Among the 97 isolates, 40 (41.2%) were classified as hvKp, and 57 (58.8%) as cKp. **Table 2** compares the distribution of capsular serotypes and virulence genes between the groups. A positive string test (hypermucoviscous phenotype) was significantly more common in hvKp strains (77.5%) than in cKp strains (19.3%, *p* < 0.05). The K2 and K20 serotypes were detected in 22.5% of hvKp isolates, with K20 significantly more prevalent in hvKp than in cKp strains (22.5% vs. 7.0%, *p* < 0.05).

### 3.3 Comparison of clinical characteristics between hvKp and cKp

Clinical data comparing hvKp and cKp infections are shown in **Table 1**. The median age of patients with hvKp infections was 70 years (IQR: 53–75 years), compared to 68 years (IQR: 54–79 years) in patients with cKp, with no significant difference (*p* > 0.05). Urinary tract infections were less common in the hvKp group than in the cKp group (25.0% vs. 35.1%, *p* < 0.05), while bacteremia was significantly more frequent in hvKp cases (15.0% vs. 3.5%, *p* < 0.05).

### 3.4 Comparison of antimicrobial resistance between hvKp and cKp

**Table 3** presents antimicrobial resistance profiles for hvKp and cKp isolates. MDR rate was significantly higher in cKp strains than in hvKp strains (52.6% vs. 22.5%, *p* < 0.05). The production of ESBLs was also more frequent in cKp strains (31.6%) than in hvKp strains (7.5%, *p* < 0.05). Significantly higher resistance to CZO (54.4% vs. 20.0%), CRO (45.6% vs. 20.0%), CIP (45.6% vs. 25.0%), LEV (33.3% vs. 10.0%), and SAM (54.4% vs. 25.0%) was observed in cKp isolates compared with hvKp isolates (all *p* < 0.05). No significant differences were observed in resistance to carbapenems or aminoglycosides between the groups.

### 3.5 Multilocus sequence typing analysis

**Figure 1:**
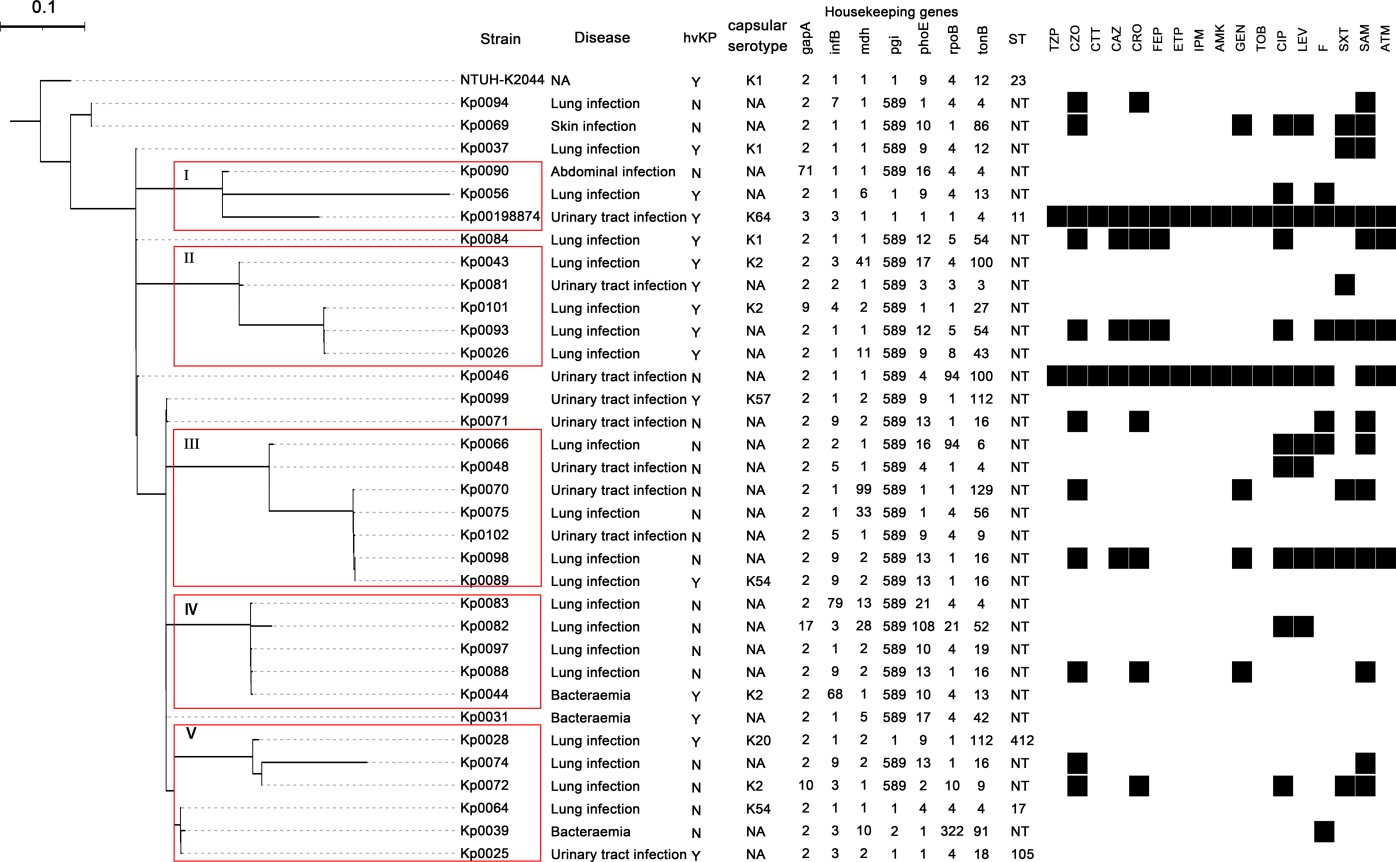
MLST dendrogram, disease, capsular serotype, and resistant phenotype of the 34 *Klebsiella pneumoniae* strains. Phylogenetic trees based on concatenated sequences of seven MLST loci using maximum likelihood methods. The strains in the red box represent clades I–V. Clades marked by squares display a highly significant bootstrap value (≥85). Abbreviations: NA, not available; ST, sequence type; NT, novel types; TZP, piperacillin–tazobactam; CZO, cefazolin; CTT, cefotetan; CAZ, ceftazidime; CRO, ceftriaxone; FEP, cefepime; ETP, ertapenem; IPM, imipenem; AMK, amikacin; GEN, gentamicin; TOB, tobramycin; CIP, ciprofloxacin; LEV, levofloxacin; F, nitrofurantoin; SXT, trimethoprim–sulfamethoxazole; SAM, ampicillin–sulbactam; ATM, aztreonam; MLST, multilocus sequence typing.

Multilocus sequence typing was conducted on 34 K*. pneumoniae* strains. A total of 70 different alleles were detected across the seven MLST loci investigated, and the *tonB* gene was the most polymorphic (20 alleles), while the *pgi* gene was the least informative locus (three alleles) (**Figure 1**). The remaining loci—*gapA*, *infB*, *mdh*, *phoE*, and *rpoB*—harbored 6, 9, 11, 12, and 9 alleles, respectively. A new allele (589) was identified in the *pgi* gene. This locus did not correspond to any specific sequence types (STs) in the global MLST database. Phylogenetic analysis grouped the isolates into five major clades (I–V), with a highly significant bootstrap value (≥85). A total of 29 distinct sequence types were identified, of which 26 were novel (**Figure 1**). Hypervirulent *K. pneumoniae* strains clustered primarily in clades I and II, whereas cKp strains were mainly found in clades III–V. High genetic heterogeneity was observed. Some sequence types appeared in multiple clades, including Kp0071, Kp0074, Kp0088, Kp0089, and Kp0098, which were distributed across four clades. Kp0093 and Kp0084 were identified in two unclassified clades.

### 3.6 Genomic features of the Kp00198874 strain

Kp00198874 was subjected to WGS. This strain was obtained from a 30-year-old female inpatient diagnosed with a community-acquired urinary tract infection. Using 33,233 reads, the shotgun sequences were assembled into one chromosome and five plasmids (pKp00198874_1–5). The chromosome had a total length of 5,445,406 bp, with an average G+C content of 57.41%. The five plasmids had lengths of 19,213, 117,195, 84,876, 11,970, and 5,596 bp, with average G+C contents of 50.43%, 54.12%, 54.07%, 51.14%, and 55.59%, respectively.

The chromosome contained approximately 5,100 protein-coding genes, 25 rRNA operons (one of which had an extra 5S rRNA gene arranged in the order 16S–23S–5S–5S, while the others followed the typical 23S–16S–5S cluster), and 85 tRNA genes. The plasmids pKp00198874_1 to pKp00198874_5 encoded approximately 203, 159, 100, 15, and 5 protein-coding genes, respectively. No rRNA or tRNA genes were identified in any plasmid.

**Figure 2:**
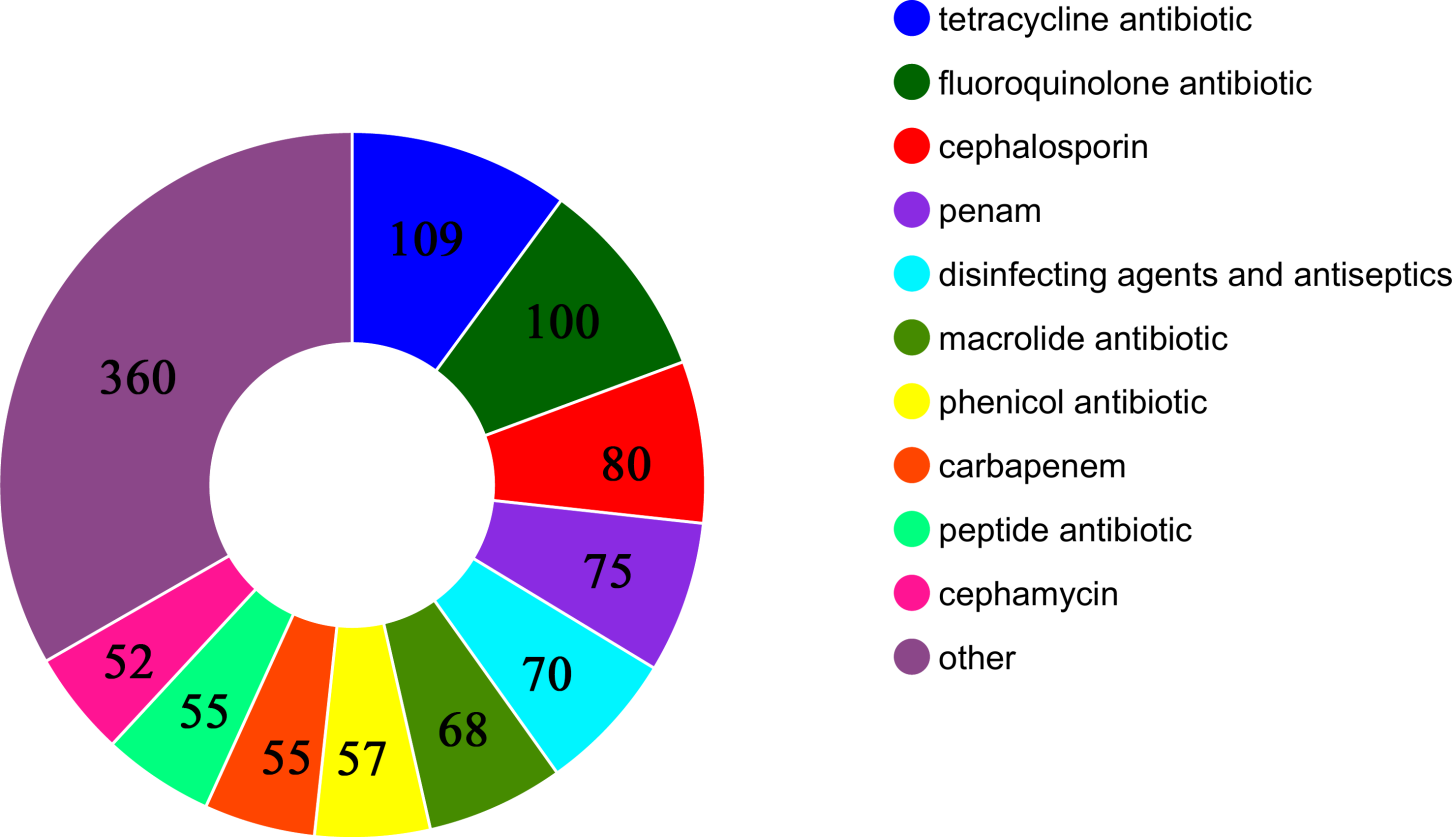
The detailed number of 1,081 antibiotic resistance genes in Kp00198874.

**Figure 3:**
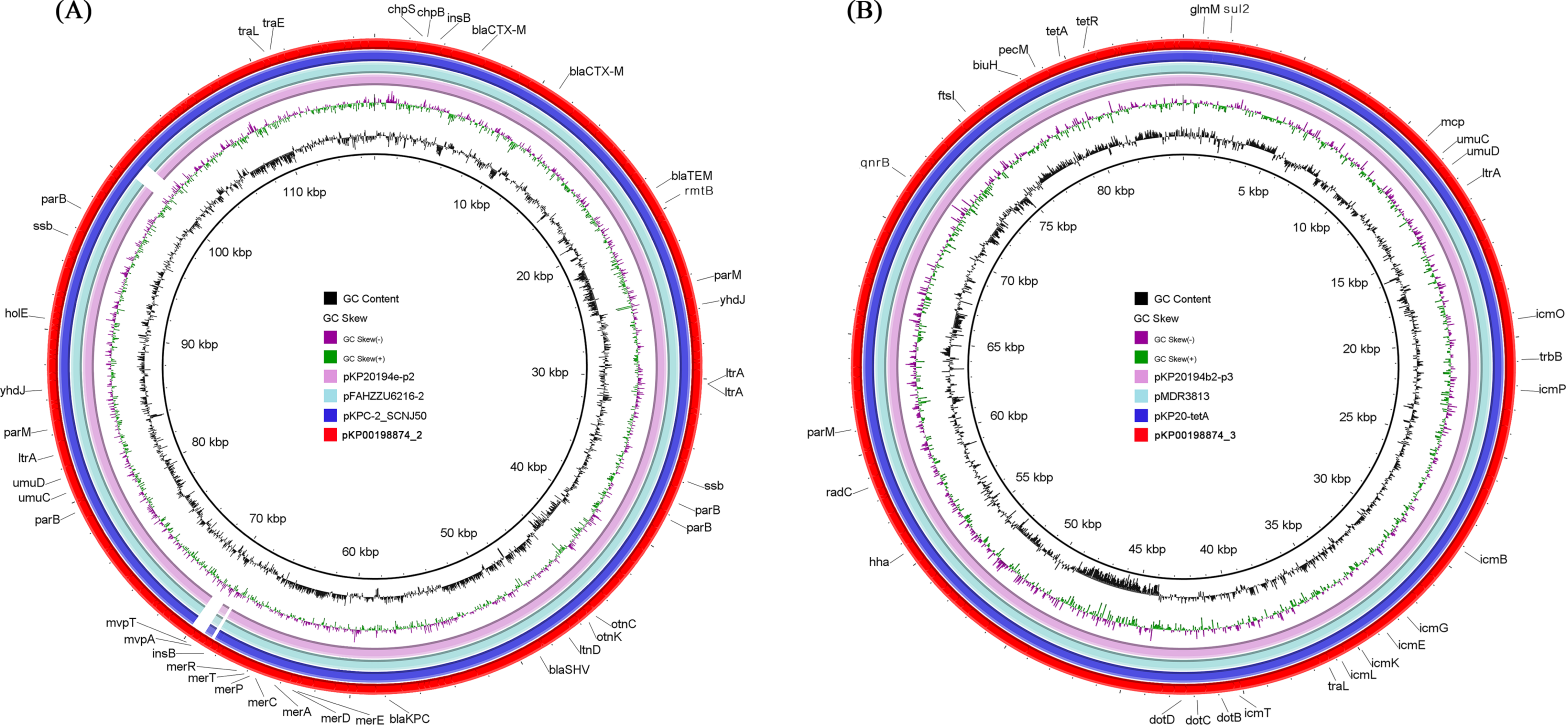
Genetic comparison of the resistance plasmids. **(A)** BRIG comparison of four plasmids, including pKP20194e-p2 from an outbreak of the ST11-K64 strain (Zhang et al., 2020); pFAHZZU6216–2 and pKPC-2_SCNJ50 exhibited a high coverage and identity with pKp00198874_2 (by BLAST). **(B)** BLAST Ring Image Generator (BRIG) comparison of four plasmids, including pKP20194b2-p3 from an outbreak of the ST11-K64 strain (Ruan et al., 2020); pMDR3813 and pKP20-tetA exhibited a high coverage and identity with pKp00198874_3 (by BLAST).

The MDR-hvKp strain Kp00198874 was identified as belonging to ST11, the dominant *K. pneumoniae* carbapenemase (KPC)-producing *K. pneumoniae* clone in China and southeastern Asia (Liu et al., 2019). The K-locus was identified as K64. Screening with CARD revealed antibiotic resistance genes (ARGs) conferring resistance to 38 classes of antimicrobial agents, including tetracyclines, fluoroquinolones, cephalosporins, penams, disinfectants, antiseptics, macrolides, phenicols, carbapenems, peptides, and cephamycins (**Figure 2**). The integration of phenotypic resistance data with genomic analysis indicated that resistance was primarily mediated by two plasmids. Plasmid pKp00198874_2 carried genes for β-lactam resistance (*blaCTX-M*, *blaTEM*, *blaSHV*, and *blaKPC*) and aminoglycoside resistance (*rmtB*), while plasmid pKp00198874_3 carried genes for fluoroquinolone (*qnrS8*), sulfonamide (*sul2*), and tetracycline [*tet(A)*] resistance. To investigate the genetic similarity of these plasmids, a blastmap comparison was conducted between pKp00198874_2 and pKp00198874_3 and similar plasmids from the NCBI GenBank database (**Figure 3**). Results showed high similarity between pKp00198874_2 and other *bla*_KPC-2_-bearing plasmids, including pKPC-2_SCNJ50 (135 kbp, 99% coverage, 99.99% identity; PP746481), pFAHZZU6216-2 (133 kbp, 98% coverage, 99.99% identity; CP182921), and pKP20194e-p2 (133 kbp, 98% coverage, 99.99% identity; CP054728). In addition, BLAST analysis showed that pKp00198874_3 shared complete homology (100% identity and coverage) with other *tet(A)*-bearing plasmids, such as pMDR3813 CP138522 (CP138522), pKP20-tetA CP154390 (CP154390), and pKP20194b2-p3 CP054765 (CP054765).

**Figure 4:**
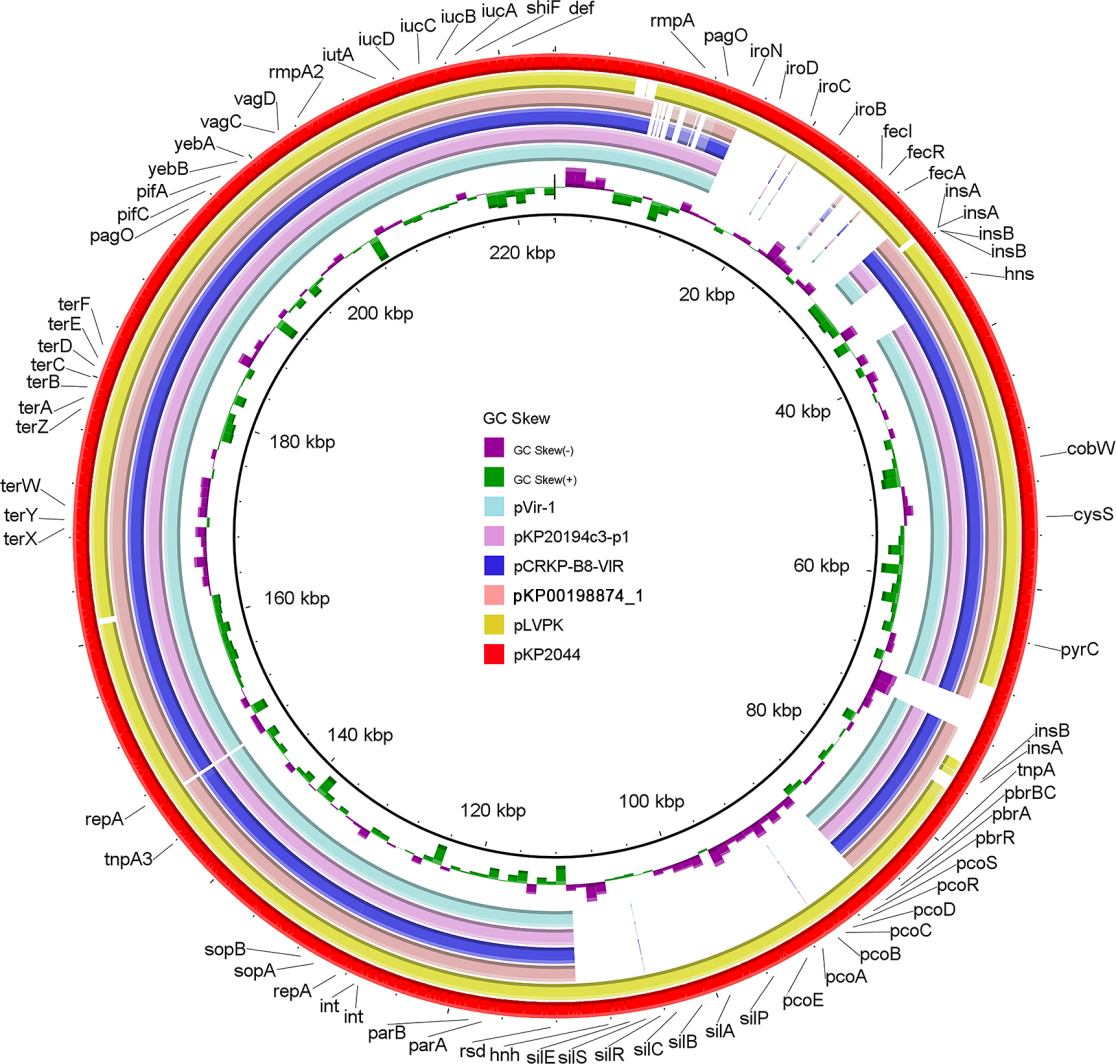
Genetic comparison of the virulence plasmid. BLAST Ring Image Generator (BRIG) comparison of six plasmids, including classical virulence plasmids pLVPK and pKP2044; pKP20194c3-p1 from an outbreak of the ST11-K64 strain (Zhang et al., 2020); pVir-1 and pCRKP-B8-VIR exhibited a high coverage and identity with pKp00198874_1 (by BLAST). pKP2044 was used as the reference plasmid.

Virulome analysis revealed several virulence genes located on the chromosome, including *fimABCDEFGHIK* (type 1 fimbriae), *mrkABCDFHIJ* (type 3 fimbriae), *iutA* (aerobactin), *fepABCDG* (ferric enterobactin), *entABCEDS* (enterobactin), *iroE* (salmochelin), and the *yersiniabactin* gene cluster comprising *ybtAEPQSTUX*, *irp1*, and *irp2*. Virulence genes *rmpA2*, *iucABCD*, and *iutA* were located on plasmid pKp00198874_1. This virulence gene distribution pattern resembled that of the classical hypervirulent strain NTUH-K2044. Results from BLAST analysis indicated that pKp00198874_1 showed high homology with other virulence plasmids, including pCRKP-B8-VIR (192 kbp, 100% coverage, 99.98% identity; CP132049), pKP20194c3-p1 (195 kbp, 98% coverage, 99.99% identity; CP054751), and pVir-1 (193 kbp, 98% coverage, 99.99% identity; CP102391). All three of these plasmids were smaller than the classical hypervirulent plasmids pKP2044 and pLVPK (**Figure 4**).

Kp00198874 harbored 27 genomic islands, ranging in size from 2,928 to 51,227 bp. Among these, five islands contained transposase- and phage-related sequences. In addition, seven intact phage-related sequences were identified, with sizes ranging from 4,725 to 53,152 bp. The genomic analysis revealed three CRISPR loci on the chromosome and five additional CRISPR loci on the plasmids.

## 4 Discussion

This study was the first to describe the clinical and molecular features of hvKp and cKp in Zhuhai City, Guangdong Province, during the COVID-19 pandemic. Recently, increasing studies have differentiated hvKp from cKp. Earlier studies primarily identified hvKp based on clinical presentation and/or a positive string test. However, Tan et al. reported that not all hvKp strains exhibit a hypermucoviscous phenotype, indicating that this classical feature may be absent in some hvKp strains (Tan et al., 2014). To date, no gold standard exists for the laboratory detection of hvKp (Yang et al., 2020). With the expanded application of WGS, several studies have confirmed that the presence of pLVPK or pLVPK-like plasmids, harboring virulence genes such as *iroBCDN*, *iucABCD*, *iutA*, and *rmpA/rmpA2*, is closely associated with hvKp strains (Hong et al., 2024; Yuan et al., 2024). More recent research has proposed that the presence of at least two of six virulence genes (*rmpA*, *rmpA2*, *iucA*, *iroB*, *peg-344*, and *peg-589*) can reliably distinguish hvKp from cKp (Yang et al., 2020)26. In the present study, 40 of 97 isolates (41.2%) met this molecular definition and were classified as hvKp. By comparison, a multicenter study conducted between February and July 2013 reported a 37.8% prevalence of aerobactin-positive hvKp strains, including 30.7% in Guangzhou (Zhang et al., 2016). A study from Dongguan People’s Hospital in 2018 reported a lower prevalence of 16.4% (Li et al., 2020). These regional variations may reflect temporal or environmental factors, including the potential impact of the COVID-19 pandemic (Liu et al., 2024). During the COVID-19 pandemic, several factors likely contributed to the increased incidence of hvKp. The widespread use of broad-spectrum antibiotics, particularly in critically ill patients, created a favorable environment for the spread of hvKp strains (Liu et al., 2024). Additionally, the increased use of immunosuppressive treatments may have compromised patients’ immune defenses, making them more susceptible to infections caused by hvKp (Mukherjee et al., 2023). Changes in infection control protocols, such as reduced surveillance and diverted resources, may have also facilitated the transmission of hvKp within hospital settings, especially in intensive care units (ICUs), which were high-risk areas for nosocomial infections (Phua et al., 2024). The vulnerability of COVID-19 patients, coupled with prolonged hospitalization and invasive procedures, likely further contributed to the rise of hvKp infections during the pandemic.

The hvKp and cKp strains also differ in their clinical and phenotypic characteristics (Russo et al., 2024). The clinical management of hvKp infections may also differ from that of cKp infections (Liu et al., 2020). In the current study, bacteremia was more frequently associated with hvKp, whereas urinary tract infections were more commonly associated with cKp. This pattern supports previous observations that hvKp is more likely to cause bloodstream infections (Lee et al., 2006). In addition, hvKp strains have been more frequently associated with community-acquired *K. pneumoniae* bacteremia than with hospital-acquired cases (Lee et al., 2006; Le et al., 2021). These findings highlight the importance of clinical vigilance for hvKp as a potential cause of bacteremia.

Capsular serotypes K1, K2, K5, K20, K54, and K57 have traditionally been linked to the hypermucoviscous phenotype and are considered indicators of hypervirulence (Jiang et al., 2024; Kubicskó et al., 2025). A study involving 230 K*. pneumoniae* isolates in China found a strong association between hvKp and serotypes K1, K2, and K20 in aerobactin-positive strains (Zhang et al., 2016). Similarly, a study from Beijing involving 159 isolates reported a significant association between hvKp and K1/K2 serotypes as defined by the presence of a combination of *rmpA*, *rmpA2*, *iucA*, *iroB*, and *peg-344* virulence genes (Liu et al., 2020). In contrast, our study found a higher prevalence of the K20 serotype among hvKp isolates, while K1 and K2 were not significantly associated. This discrepancy may be attributable to differences in hvKp definitions and classification methods across studies.

The clinical significance of *K. pneumoniae* continues to increase owing to the emergence of MDR variants and the growing threat posed by hvKp as a high-risk pathogen (Alharbi et al., 2023). Previously, most hvKp strains, often identified using string testing, were susceptible to antimicrobial agents. However, with increasing antibiotic use and selective pressure, MDR-hvKp strains have emerged as serious threats, capable of causing severe and difficult-to-treat infections (Tang et al., 2020). Mortality rates for MDR-hvKp infections in China have been reported to range from 56.3% to 66.7% (Tang et al., 2023).

In the present study, antimicrobial susceptibility testing showed that hvKp strains were more susceptible to several antibiotic classes, including SAM, CZO, CRO, and CIP, with susceptibility rates ranging from 10.0% to 25.0%. The MDR rate among hvKp strains (22.5%) was significantly lower than that of cKp strains (56.63%), and ESBL production was also less frequent in hvKp strains (7.5%) compared with cKp strains (31.58%). However, carbapenem resistance rates were similar between hvKp and cKp strains. These findings are consistent with previous reports from China (Liu and Guo, 2019; Li et al., 2020; Yang et al., 2020). In a study conducted in Hebei Province, 20.0% (16/80) of hvKp isolates were identified as MDR between 2008 and 2014, and the proportion of MDR-hvKp strains has continued to rise (Liu and Guo, 2019). A multicenter study also reported an increase in MDR-hvKp prevalence in China from 2012 to 2017 (Gao et al., 2020; Tang et al., 2023). A recent study has suggested that the overuse of antimicrobials and a growing number of critically ill patients in overcrowded ICUs likely contributed to the emergence and spread of MDR organisms during the COVID-19 pandemic (Phua et al., 2024). During the same period, the prevalence of MDR-hvKp increased across multiple sequence types and geographic regions, highlighting the need for ongoing genomic surveillance and early detection of emerging variants (Liu et al., 2024). Interestingly, a cKp strain Kp0046 was identified as sharing a highly similar resistance pattern with the ST11-K64 hv-CRKp strains Kp00198874, but exhibiting distinct patterns in MLST and virulence factors. The Kp0046 strain has been submitted to gene-sequencing companies for WGS analysis. Further detailed research will be conducted, and the findings will be incorporated into subsequent publications.

To further characterize the population structure, MLST analysis was performed to assess the phylogenetic relationships among the 34 K*. pneumoniae* strains. These isolates were classified into 29 distinct sequence types, indicating low genetic homology among them. In addition, strains with identical sequence types (e.g., Kp0071, Kp0074, Kp0088, Kp0089, and Kp0098) were found across different phylogenetic clades, indicating that they are not clonal. This implies that the isolates likely represent sporadic infections, independent transmission events, or localized outbreaks. In this study, two new STs were identified in Kp0039 (MLST profile: 2, 3, 10, 2, 1, 322, and 91) and Kp0056 (MLST profile: 2, 1, 6, 1, 9, 4, and 13), each differing by one locus from ST8124 (MLST profile: 2, 40, 10, 2, 1, 322, and 91) and ST592 (MLST profile: 2, 3, 6, 1, 9, 4, and 13), respectively. This means that these strains may have originated from the same clonal group. Interestingly, most of the novel STs share a common feature: their *pgi* gene is allele 589. This locus did not correspond to any specific STs in the global MLST database. This finding indicates that these new STs may share a common evolutionary history or that those isolates have acquired certain specific metabolic advantages under certain hospital environmental conditions. Our data show high genetic diversity in our isolates, suggesting that the global population structure of *K. pneumoniae* is still poorly understood. Future studies should be focused on the dynamics of *K. pneumoniae* spread and evolution.

Although carbapenem-resistant *K. pneumoniae* strains are not typically hypervirulent, cases of hv-CRKp have become increasingly prevalent in mainland China (Zhang et al., 2020). These strains may arise via two main pathways: acquisition of a carbapenemase-encoding plasmid by an hvKp strain (typically ST23 with K1/K2 capsule) (Dong et al., 2019; Shen et al., 2019) or acquisition of a pLVPK-like virulence plasmid by a carbapenem-resistant *K. pneumoniae* strain (often ST11) (Ruan et al., 2020; Yang et al., 2020). Our MDR isolate Kp00198874 likely represents the latter mechanism, as it was identified as an ST11-K64 strain carrying a pLVPK-like plasmid. pKp00198874_1 and pLVPK share several homologous regions, both containing virulence factors such as *rmpA2*, *iutA*, and *iucABCD*, which contribute to the hypervirulent phenotype of hvKp strains. These factors enhance the strain’s ability to cause severe infections, particularly in immunocompromised hosts. This suggests that the pKp00198874_1 plasmid may have evolved from similar genetic elements spread through horizontal gene transfer. In addition to its virulence plasmid, the Kp0019887 strain also carries two distinct resistance plasmids, which harbor several resistance genes, including β-lactam resistance [*blaCTX-M*, *blaTEM*, *blaSHV*, *blaKPC*, *qnrS8*, *sul2*, and *tet(A)*]. The phenotypic and genotypic characteristics observed in Kp00198874 are consistent with those in the ST11-K64 hv-CRKp strains identified in other studies (Zhang et al., 2020; Zhou et al., 2021; Wei et al., 2022). The coexistence of virulence and resistance plasmids within the ST11-K64 hv-CRKp enhances its ability to survive in hostile hospital environments, contributing to the increasing prevalence of hv-CRKp strains globally. One study documented inter-hospital transmission of 13 ST11-K64 hv-CRKp isolates, highlighting this clone’s role as a competent host for virulence plasmids and a potential driver of future outbreaks (Zhang et al., 2020). Furthermore, recent studies on CRKp epidemiology in China have shown that the ST11-K64 CRKp clone predominates in clinic settings and accounts for over 50% of isolates in various reports (Liu et al., 2023; Spadar et al., 2023). The emergence of ST11-K64 hv-CRKp poses a significant threat to clinical care, emphasizing the urgent need for active surveillance and containment strategies.

The present study has several limitations. First, the study was conducted at a single hospital, which may limit its representativeness and prevent cross-hospital genomic comparisons. Second, due to the retrospective design and the diversity of patient sources (including both inpatients and outpatients), only limited clinical data and patient outcomes were available. Third, the MLST and WGS of all strains have not been analyzed. Future work is warranted to invite more hospitals in Guangdong to participate in this research and collect more isolates to explore their characteristics among different hospitals.

In summary, the prevalence of hvKp in Guangdong Province appears to have increased during the COVID-19 pandemic. Hypervirulent *K. pneumoniae* strains were more frequently associated with bacteremia than cKp strains, and the convergence of hypervirulence and multidrug resistance, particularly in ST11-K64 hv-CRKp, raises serious public health concerns. Ongoing surveillance, rapid molecular diagnostics, and robust infection control measures are essential to monitor and mitigate the spread of these high-risk pathogens.

## Data Availability

All whole-genome sequencing data from the study are available in the National Center for Biotechnology Information (NCBI) under project accession number PRJNA1248222. Fully de-identified clinical data are available from the corresponding author on reasonable request and subject to ethical approval.
