# Supplementary material for: Epidemiological and characteristic differences of hypervirulent and classical Klebsiella pneumoniae: a clinical and genomic study in Southern China during the COVID-19 pandemic
Source: Front Cell Infect Microbiol. 2026 Jan 16;15:1701929. doi: 10.3389/fcimb.2025.1701929 (PMC12855528; doi:10.3389/fcimb.2025.1701929)
Supplement: Supplementary file 1 [file Table1.docx]

**Supplementary**

Table S1. Primers used in this study.

| **Genes** | **Primer^a^ (5’-3’)** | **Annealing temperature (℃)** | **Size (bp)** |
| --- | --- | --- | --- |
| **Virulence genes** | | | |
| *iuc*A | F1:AATCAATGGCTATTCCCGCTG R1:CGCTTCACTTCTTTCACTGACAGG | 59℃ | 239 |
|  | F2:GCTTATTTCTCCCCAACCC R2:TCAGCCCTTTAGCGACAAG | 59℃ | 583 |
| *iro*B | F1:ATCTCATCATCTACCCTCCGCTC R1:GGTTCGCCGTCGTTTTCAA | 59℃ | 235 |
|  | F2:CAAAAAAGCAGCAGAGGC R2:TCACTGGCGGAATCCAACAC | 59℃ | 585 |
| *peg*-344 | F1:CTTGAAACTATCCCTCCAGTC R1:CCAGCGAAAGAATAACCCC | 53℃ | 508 |
|  | F2:AAAGGACAGAAAGCCAGTG R2:CAATGACGAGGGGGATAATC | 53℃ | 411 |
| *peg*-589 | F1:TGAACCCCTGAAGGTCTATC R1:GTGATGAATAAACTACTGCGGC | 55℃ | 236 |
|  | F2:GGACCAGAAAACACAGGA R2:AGCGGCGATTTCTTCTC | 55℃ | 124 |
| *peg*-1631 | F1:GGGATTTATCAACCGCTTTG R1:TCTCCAGCATCATCGTCA | 59℃ | 503 |
|  | F2:TTGTGGCAGAAGACCCG R2:TCTCCAGCATCATCGTCAG | 59℃ | 486 |
| prmpA | F:GAGTAGTTAATAAATCAATAGCAAT R:CAGTAGGCATTGCAGCA | 50℃ | 332 |
| prmpA2 | F:GTGCAATAAGGATGTTACATTA R:GGATGCCCTCCTCCTG | 50℃ | 430 |
| **Capsular Serotype** | | | |
| K1 | F:GGTGCTCTTTACATCATTGC | 50℃ | 1283 |
|  | R:GCAATGGCCATTTGCGTTAG |  |  |
| K2 | F:GACCCGATATTCATACTTGACAGAG | 52℃ | 641 |
|  | R:CCTGAAGTAAAATCGTAAATAGATGGC |  |  |
| K5 | F:TGGTAGTGATGCTCGCGA | 53℃ | 280 |
|  | R:CCTGAACCCACCCCAATC |  |  |
| K20 | F:CGGTGCTACAGTGCATCATT | 54℃ | 741 |
|  | R:GTTATACGATGCTCAGTCGC |  |  |
| K54 | F:CATTAGCTCAGTGGTTGGCT | 52℃ | 881 |
|  | R:GCTTGACAAACACCATAGCAG |  |  |
| K57 | F:CTCAGGGCTAGAAGTGTCAT | 50℃ | 1037 |
|  | R:CACTAACCCAGAAAGTCGAG |  |  |
| **Housekeeping genes** | | | |
| *phoE* | F:GTTTTCCCAGTCACGACGTTGTAACCTACCGCAACACCGACTTCTTCGG  R:TTGTGAGCGGATAACAATTTCTGATCAGAACTGGTAGGTGAT | 50℃ | 420 |
| *gapA* | F:GTTTTCCCAGTCACGACGTTGTATGAAATATGACTCCACTCACGG  R:TTGTGAGCGGATAACAATTTCCTTCAGAAGCGGCTTTGATGGCTT | 50℃ | 450 |
| *infB* | F:GTTTTCCCAGTCACGACGTTGTACTCGCTGCTGGACTATATTCG  R:TTGTGAGCGGATAACAATTTCCGCTTTCAGCTCAAGAACTTC | 50℃ | 318 |
| *mdh* | F:GTTTTCCCAGTCACGACGTTGTACCCAACTCGCTTCAGGTTCAG  R:TTGTGAGCGGATAACAATTTCCCGTTTTTCCCCAGCAGCAG | 50℃ | 477 |
| *pgi* | F:GTTTTCCCAGTCACGACGTTGTAGAGAAAAACCTGCCTGTACTGCTGGC  R:TTGTGAGCGGATAACAATTTCCGCGCCACGCTTTATAGCGGTTAAT | 50℃ | 432 |
| *rpoB* | F:GTTTTCCCAGTCACGACGTTGTAGGCGAAATGGCWGAGAACCA  R:TTGTGAGCGGATAACAATTTCGAGTCTTCGAAGTTGTAACC | 50℃ | 501 |
| *tonB* | F:GTTTTCCCAGTCACGACGTTGTACTTTATACCTCGGTACATCAGGTT  R:TTGTGAGCGGATAACAATTTCATTCGCCGGCTGRGCRGAGAG | 50℃ | 414 |

^a^ F, forward primer; R, reverse primer.
